# New products

**DOI:** 10.1155/S1463924689000222

**Published:** 1989


Journal of Automatic Chemistry Vol. 11, No. 2 (March–April 1989), pp. 91–97

### New products

### Chemical information retrieval: an intelligent text retrieval package

Harwell Computer Power has launched a text information retrieval system for the chemical industry which incorporates two major breakthroughs in technology.

STATUS/IQ is based around STATUS, originally developed by the Harwell Laboratory, but can understand questions addressed to it in plain, standard English and can assess the relative importance of each piece of retrieved text.

Traditional free text systems allow users to ask questions via a command or menu-driven interface that simply searches on any word. However, STATUS/IQ's natural language ability allows the user to ask questions such as:

'What are the safety standards that control the manufacture of isocyanates for paint?'

STATUS/IQ will immediately recognise the concepts in the sentence such as 'safety standards,' 'manufacture,' 'isocyanates' and 'paint.' STATUS will then search through the database to find every relevant article.

The second technological breakthrough – the ranking of data in order of relevance – is in some ways a more fundamental advance in information technology.

Whereas traditional systems will often retrieve several hundreds of articles that include specified keywords, STATUS/IQ finds all of the information and indicates which pieces are most relevant to your question.

Both STATUS and STATUS/IQ automatically index every word in each document entered into the database, and allow the location of words, phrases or concepts. STATUS/IQ, which uses natural language rather than keying search words into menu options, virtually eliminates the need for training and can be used by staff with little or not computer skills.

*For further information, contact: Harwell Computer Power, Curie Avenue, Harwell, Oxfordshire OX11 0QW, UK.*

### Personal computer controls dissolution testing

The automatic set-up and control of tablet dissolution testing with the HP 8450A diode array UV/vis spectrophotometer can now be simplified by using HP's PC-compatible Vectra computer as the system controller.

This new dissolution testing package for pharmaceutical laboratories, comprising HP Vectra, HP 8450A spectrophotometer, accessories and software, provides both single and multi-component analysis using proven mathematical algorithms. Methods may be stored for future use.

Up to three six-vessel dissolution baths may be used simultaneously, and a control sample for each can be measured automatically to validate system performance before the test. During testing, measurements are made at constant intervals or at user-specified times. Automatic pump control is available for long term tests.

Up to five quality control specifications can be defined, and results from any vessels that do not meet these are listed separately.

Data from up to four baths may be combined in one report, which is produced in the format required by the user. Data are stored as ASCII files, and can thus be imported into other programs. Complete statistical analyses are included in the report.

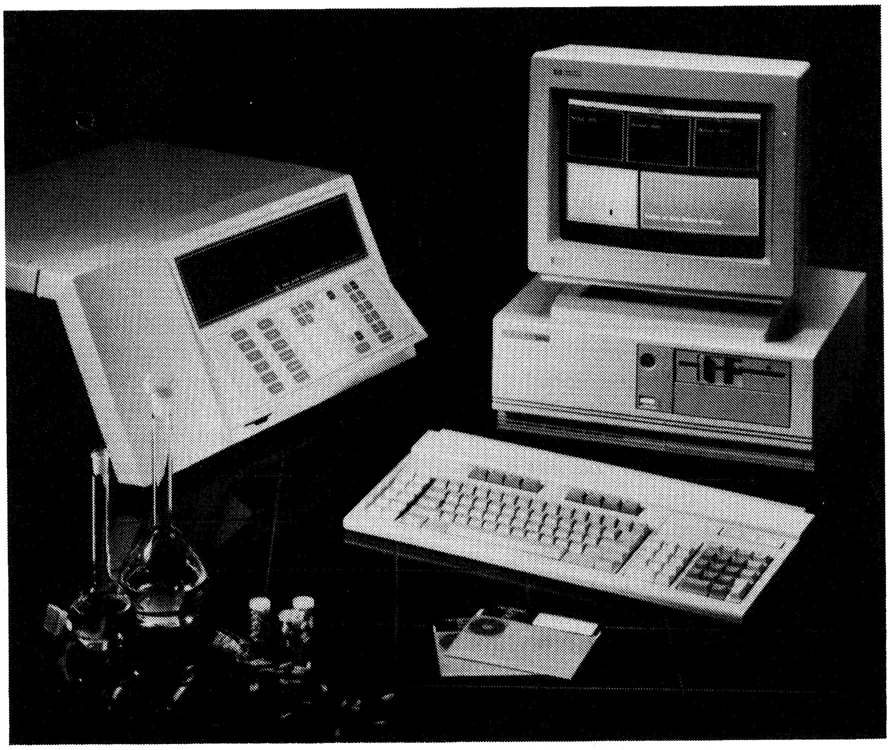

*Dissolution-testing system from Hewlett-Packard, consisting of an HP8450A diode-array UV/visible spectrophotometer, the HP89024B dissolution testing kit and the system controller, an HP Vectra PC.*

0142–0453/89 $3.00 © 1989 Taylor & Francis Ltd.

*For further information, contact: Analytical Products Group, Hewlett-Packard Limited, Miller House, The Ring, Bracknell, Berks. RG12 1XN, UK. Tel.: 0344 424898.*

### Quantitation and enzyme kinetics software for diode array spectrometry

New software just released by Hewlett-Packard for the UV/vis ChemStation further extends the capabilities of the HP 8452A diode array UV/vis spectrophotometer.

Quantitative analysis is greatly facilitated by the HP 89511A quantitation software package, designed to aid the development of quantitative methods and multicomponent analysis. The new software takes full advantage of the advanced diode array technology of the HP 8452A.

For single component analysis, the software allows the selection of wavelengths which provide the best calibration curve and the greatest reproducibility, and the determination of sample impurities. In multicomponent analysis, the software allows concentrations of up to 12 components to be determined within a known sample matrix, allows the use of absorption and derivative spectra and saves time in method development by mathematically synthesizing spectra prior to measuring actual samples so that the feasibility of multicomponent analysis may be determined quickly.

A new enzyme kinetics package, HP 89512A, provides biochemists with the means to investigate enzyme reactions, rate data and reaction models with unmatched speed and accuracy. The combination of diode array scanning of the whole wavelength range with the fast data evaluation of this new software allows spectral information to be stored and evaluated post-run. In this way the design of subsequent experiments may be optimised after the initial run, eliminating repetitive trial and error.

Reaction rate data may be determined quickly and easily with various types of curve fit. Nine mathematical models for enzyme reactions provide flexibility; alternatively users can develop models from their own equations. The kinetics of several reactions may be measured in parallel with the help of a new multi-cell transport mechanism for the HP 8452A.

New full-colour, illustrated brochures are available for both packages. The brochures describe the software in detail, and contain application examples.

The brochures are available free of charge from Hewlett-Packard.

*For further information, contact: Hewlett-Packard Limited, Miller House, The Ring, Bracknell, Berks. RG12 1XN, UK, Tel.: 0344 424898.*

### Automated data collection using single computer

The Moncal 10 system software with an IBM personal computer collects test results from Monsanto Rheometer, Mooney and Tensometer 10 instruments.

The system collects the results from as many as 10 instruments for recall display or printing. Results print automatically on detection of a limits violation. Other test data may be input manually. The user may also reformat his files for use with other commercial software.

The Moncal 10 system compares specification limits with test data for each instrument. On detection of a limit violation, the Moncal 10 system automatically displays a visual alarm and illuminates the failed light at the test instrument. Any limit violation is stored with the data so that reports may be made with limits applicable at the time of test. The system is capable of storing 1000 sets of compound limits.

For each test the running statistics (average and standard deviation) are automatically updated. They can be reset to start a new series. This gives an indication of the position (average) and the variation (standard deviation) over a fixed period of time. Off-line reports recalculate the average and standard deviation over a defined time and/or batch range.

Further statistical analysis includes process capability (Cp, CpK) and histogram display for results comparison with the normal curve and control limits.

The batch report function gives a compilation of all tests carried out for any specific batch. This includes pass or fail messages.

As many as five material properties can be entered via the keyboard (i.e. hardness, SG).

Manual input data is processed in an identical way to automatically collected data.

Moncal 10 requires an IBM personal computer with 20 MByte hard disk, a floppy disk and HPIB board. Screen entry facilities, prompt messages and function keys simplify system operation.

The Moncal 10 system accepts data directly from the test instruments or via the keyboard. Data from Monsanto Rheometer, Mooney and Tensomer 10 is directly transmitted through unique interfaces having IEEE488 capability available from Monsanto.

*For further information, contact: Monsanto plc, Edison Road, Dorcan, Swindon, Wiltshire SN3 5HN, UK. Tel.: (0793) 31315.*

### Kodak Ekta chem analyzers: new brochure

A 6-page free brochure, *The Configurable Analyzers from Kodak,* describes the modular Kodak Ektachem 700 analyzers and the desktop Ektachem DT system. The modular design of these systems means that new testing and sample management capabilities can be added at any time, maintaining flexibility and avoiding obsolescence.

*For further information, contact: Eastman Kodak Co., Dept. 412 L, 343 State Street, Rochester, NY 14650, USA. Tel.: 800 445 6325.*

### Oxygen analyser for laboratory use

The C5A oxygen analyser from Kane-May is a simple to use, rugged, portable meter which, at £225·00, offers an economic and convenient method of measuring oxygen. The C5A features a LED display, re-usable filter trap and a retractable stainless-steel probe, and comes complete with carrying case and instructions. For flue gas measuring applications, fuel efficiency charts are supplied as standard.

*For further information, contact: Kane-May Ltd., Swallowfield, Welwyn Garden City, Hertfordshire AL7 1JP, UK. Tel.: 0707 331202.*

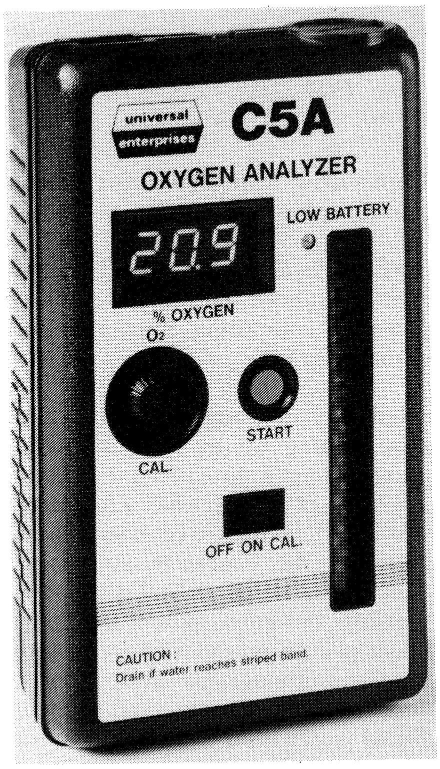

*CSA oxygen analyser from Kane-May Ltd.*

### On-line thermal conductivity analyser

Teledyne's Model 235 Thermal Conductivity (T/C) Analyser provides accurate on-line monitoring of gases in a variety of applications.

Typical applications for the Model 234 include: hydrogen analysis in petroleum refinery hydrocarbon streams, ammonia synthesis, turbine generators and blanketing gases; gas purity monitoring of argon, oxygen, hydrogen, nitrogen, helium and other gases in air liquefaction; monitoring gas proportions for process control of mixtures such as proportions for process control of mixtures such as $N_2$/argon, $H_2/N_2$, helium/air and $O_2/N_2$; and other applications where the thermal conductivity of one gas is significantly different from that of another gas (or gases) in the mixture being monitored.

The Model 235 operates by comparing the amount of heat absorbed by the sample gas with that absorbed by a known reference gas. The difference is electronically analysed with a simple Wheatstone bridge. The result is provided as a signal output.

The Model 235 features electronic temperature control of the T/C cell for optimum accuracy and stability. This 2-filament nickel-plated brass T/C cell requires only a simple periodic calibration to provide many years of service. The cell is the heart of the Model 235 and has proven its reliability, durability and accuracy in over 20 years of field use.

Also available with the Model 235 are alarms, meter readouts, integral gas control panels, MADC outputs and panel-mounted or explosion-proof enclosures. Standard configurations are also available that utilize a separate control unit for mounting in control rooms or other remote locations.

*For further information, contact: Teledyne Analytical Instruments, The Harlequin Centre, Southall Lane, Southall, Middlesex UB2 5NH, UK. Tel.: 01 571 9596.*

### TA4000 Thermal analysis system

The Mettler thermal analysis system is based on the modular concept. It comprises measuring cells for DSC, TMA and TG. The experimental data can be evaluated either in the TA TC11 processor or on an attached personal computer with the TA72 software. A Mettler analytical or microbalance can be attached via the built-in CL interface; weight values can thus be transferred automatically.

The hub of the TA4000 system is the newly designed TA TC11 processor. It contains the complete software in the basic version, method memory for 10 standard methods, and evaluation programs stored as standard. Automatic calibration routines and automatic, intelligible scaling of the plots are also built into the TC11.

All components of the system (measuring cells, balance, plotter personal computer) can be attached to the standard interfaces. If different methods are in use, the measuring cell selector switch allows facile selection of the desired measuring cell.

For a temperature range spanning −170 °C to 750 °C various DSC measuring cells all based on the same measurement principle are available. The DSC measuring cells differ, however, in their effective temperature range, construction and shape thereby ensuring optimum management of a wide range of analytical problems.

The new DSC25 measuring cell measures the heat flow in the effective temperature range −20 °C to 750 °C. A sophisticated mechanism for the opening and closing of the furnace chamber makes insertion even simpler. With the DSC25 measuring cell, decompositions of, for instance, high temperature resistant plastics can be investigated effortlessly and accurately.

*For further information, contact: Mettler Instrumente AG, CH-8606 Greifensee, Switzerland. Tel.: 01 944 2872.*

### New colour brochure on gas analysis systems

Spectramass Ltd. have announced the publication of a new brochure describing their state of the art COMPASS systems for precision gas analysis. This 4-page colour glossy is a valuable information source aiding the selection of gas analysis systems for a wide range of applications, including environmental pollution monitoring, chemical process control, pharmaceutical quality assurance and residual gas analysis in high vacuums.

*For further information, contact: Spectramass Ltd., Radnor Park, Congleton, Cheshire CW12 4XR, UK. Tel.: 0260 279531.*

### A family of electrolyte analysers

The Ciba Corning 600 Series of clinical electrolyte analysers combines advanced sensor technology with minimum maintenance requirements. Units in the range include the 614, 634, 644 and the 664/Fast Four analyser. All can carry out measurements from serum, plasma, whole blood or urine.

All models in the 600 series utilise an easy to use interactive display which guides the user through every operation. A series of questions, prompts and statements are displayed in any of the five different languages available. Simple instructions guide the operator through the programmed maintenance procedures.

The 614 Na/K and 634 Ca/pH analysers are ideal for paediatric and foetal applications, and can accept samples as small as 35 μl. Other options availabled with the 634 include the ability to report ionised calcium at any pH of the sample, corrected to the pH of 7.4, or the patient's *in vivo* pH.

The 644 $Na^+$, $K^+$, Cl and 664/Fast Four $Na^+$, $K^+$, $Cl^-$, $t_{CO2}$ analysers are more suitable for laboratories which have a larger throughput of samples. The 664 is capable of a production rate of 400 results per hour.

Sample batches can be interrupted by a STAT sample; results are available in 75 s. The 664 will automatically return back to the sample requiring analysis when the STAT sample was introduced.

This family of electrolyte analysers caters for all needs: the 614 and 634 are suitable for laboratories with a high number of priority samples but a lower sample throughput whereas the 644 and 664 Fast Four are ideal for larger sample throughput laboratories which still need to run a few priority samples.

*For further information, contact: Ciba Corning Diagnostics Ltd., Halstead, Essex CO9 2DX, UK. Tel.: 0787 472461.*

### Complete blood gas analysis systems

A complete support package of quality control materials, sampling devices and data management systems now complements the Ciba Corning 200 series of blood gas analysers. Extending this 'system concept' are remote control and monitoring facilities and the option of linkage to co-oximetry for oxygen dissociation curve analysis.

The 278 directly measures pH, $pCO_2$, and $pO_2$, calculates bicarbonate (actual or standard) base excess (*in vivo* or *in vitro*), $tCO_2$, oxygen saturation, oxygen content and capacity (with haemoglobin input), and also the $AaDO_2$ and a/A ratio (with $FIO_2$ input). The model 280 measures the clinically significant total haemoglobin value. At the top of the new range, the model 288 includes sodium, potassium and a choice of ionised calcium or chloride with all the above parameters. The anion gap is also calculated.

The 200 series electrodes have been designed to eliminate the time consuming membrane change procedures required by other electrodes. They have an extended life, enabling the operator to run samples uninterrupted by the need to change membranes. All electrodes now serve a dual purpose; as sensors to detect analytes present in the sample, and as the actual sample pathway. The response time for the electrode endpoint on all electrodes is approximately 45–60 s.

Both the model 288 and 280 measure total haemoglobin using a flow injection analysis method to give correlation with haematology equipment. Thirteen microlitres of whole blood are diluted inside the 288/280 by mixing it with haemoglobin reagent in a special mixing block. This zigzag mixing chamber provides a fast reaction time (15 s) without clotting as the reagent/blood ratio is very high. The total system is very fast (typically 45 s), is self-cleaning and, due to the composition of the haemoglobin reagents, will measure turbid samples with total accuracy.

This facility of total haemoglobin measurement means that in emergency situations, samples do not have to be despatched to a haematology laboratory. Anaesthetists can therefore get a quick and accurate picture of the true oxygen carrying capacity of the blood.

Software design is such that the most frequently accessed functions are available with a minimum of key operation, and a quick and easy return to menu. This is characterised by the 'Ready, Introduce Sample' message. Features which can be customised include QC and patient reference ranges, programmable calibration intervals and printing options. Built-in memory storage allows recall of results and tracking of QC performance.

*For further information, contact: Ciba Corning Diagnostics Ltd., Halstead, Essex CO9 2DX, UK. Tel.: 0787 472461.*

### Computing integrator for chromatography

Spectra-Physics Autolab Division has announced ChromJet, a fast, powerful computing integrator for chromatography.

In addition to processing, storing and reintegrating up to 128 chromatograms using a patented integration algorithm, ChromJet has a full-sized QWERTY computer keyboard and a four-line, 40 character supertwist backlighted liquid crystal display. A specially designed (patent pending) inkjet printer provides clear, easy to read chromatograms and reports, and accommodates convenient Z-fold paper from either the front or the back of the instrument.

ChromJet provides low noise, high linearity, high speed data integration, up to 60 slices per second, and ensures accuracy even with fast capillary GC or high speed LC signals. ChromJet includes features such as drawn-in baselines, baseline subtraction, batch re-processing, built-in statistics, and time functions. A dual channel version is available, as well as optional extended BASIC programming and extra memory (to 512 Kbytes).

ChromJet is available from Spectra Physics starting at £1800.

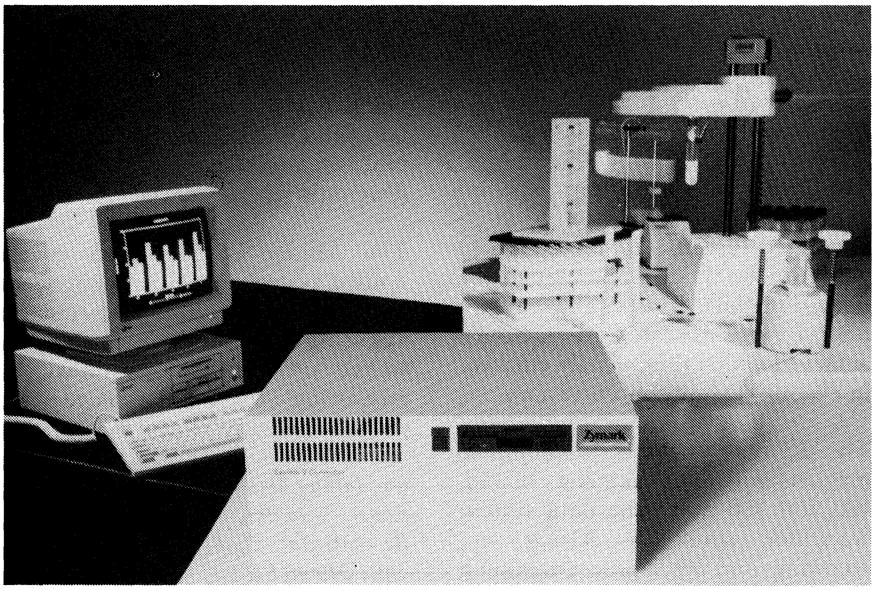

*Zymate laboratory robotics system controller from Zymark.*

*For further information, contact: Spectra-Physics Autolab Division, Boundary Way, Hemel Hempstead HP2 7SH, UK. Tel.: 0442 232322.*

### Zymate laboratory robotics system controller

Zymark's System V Controller is an advanced architecture Zymate laboratory robotics system controller which can operate directly with MS-DOS based personal computers. The System V controller is fully compatible with existing Zymate modules and EasyLab application programs but provides up to three times faster EasyLab processing and has three times the dictionary capacity of conventional Zymate controllers. PC compatibility allows ease of data management and report writing as well as archiving and transmission to central computers.

*For further information, contact: Zymark Corporation, Zymark Center, Hopkinton, MA 01748, USA. Tel.: 508 435 9501.*

### Ion concentration measurement integrated with titration

By simply plugging in the VIA103 Direct Ion Concentration Module, the powerful TitraLab High Performance Titration Laboratory can perform ISE measurements under standardized conditions with respect to electrode stability and waiting time. Together with the automatic addition of an auxiliary reagent, e.g. an ion strength adjuster, this means excellent reproducibility. The best possible accuracy throughout the electrodes' wide measuring range is obtained by calibration with up to five standard solutions.

The calibration data of each electrode are stored together with other method parameters in a memory covering up to 60 methods. The method parameters include automatic selection of electrode input and burette position. To change from one method to another you just key in the method number, and your TitraLab system is ready to go – safe and convenient.

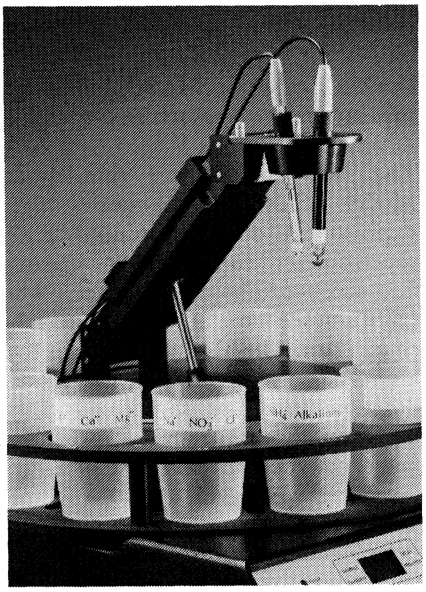

*Ion concentration measurement from Radiometer Analytical.*

In order to reduce operator costs, TitraLab and VIA103 Direct Ion Concentration Module offer full automation with a SAC80 Sample Changer. The up to five-point calibration can be performed automatically including reagent addition. On samples in the 20 position Sample Changer, several different ISE measurements can be performed in combination with pH or conductivity measurements and/or all types of titrations.

*For further information, contact: Radiometer Analytical A/S, 49 Krogshojvej, DK-2880 Bagsvaerd, Denmark. Tel.: 45 1 696311.*

### Automated DNA sequencing

An applications publication from Beckman explains how the Biomek 1000 automated laboratory workstation can be used to automate DNA sequencing according to the dideoxy method of Sanger and Coubon.

Full details are provided on the methodology, which has been developed by Beckman, including pipetting patterns used for template isolation in a 96-well plate. There are also details of the Biomek 1000 itself.

Biomek is particularly effective for automating DNA sequencing due largely to its ability to pipette 2 µl volumes with a fine precision. The recent introduction of a new eight-channel pipetting tool for Biomek has added considerable speed to the reagent addition steps in Biomek's operation.

*For further information, contact: Beckman, Progress Road, Sands Industrial Estate, High Wycombe, Bucks, UK. Tel.: 0494 41181.*

### Automatic alcohol analyser

Skalar Analytical have introduced the $SAN^{plus}$ generation of segmented flow analysers for the automatic determination of ethanol in alcoholic beverages. The $SAN^{plus}$ analyser can determine up to 30 samples per hour, from low alcohol beverages to samples with 30–40% alcohol.

Samples are introduced automatically into the analyser in which they are diluted, distilled (to overcome matrix problems), resampled and mixed with potassium bichromate. The $Cr^{6+}$ is reduced in the presence of ethanol to $Cr^{3+}$, the green complex of which is determined spectrophotometrically at 600 nm.

The ethanol analyser consists of: SA1000 sample charger, SA2005 peristaltic pump, SA module holder, SA01205007 chemistry module, SA6250 matrix photometer and SA8606 data system. The SA7011 single channel chart recorder and SA1500 rinsing valve are optional.

Other analyses automated on the segmented flow analyser for the brewing industry are: bitterness, colour, pH, free amino nitrogen, polyphenols, diacetyl, sulphur dioxide, carbonate and thiobarbituric acid number. For the wine industry Skalar has automated: acetic acid, acidity (total), chloride, density, ethanol, glucose, glucose/fructose, glucose/fructose/saccharose, glycerol, hydroxymethylfurfural, iron, lactic acid, malic acid, nitrate, pH, phosphate, reducing sugars (total), sulphur dioxide (free), sulphur dioxide (total), tartaric acid and volatile acids.

For alcohol-free and low alcohol bears, Skalar has automated the enzymatic method specified in 'EBC method 9.2' and in 'methods of enzymatic food analysis' (Boehringer Mannheim).

*For further information, contact: Skalar Analytical, P.O. Box 3237, 4800 De Breda, The Netherlands. Tel.: 76 22 54 77.*

### High sensitivity electron spin resonance

Jeol have released a new range of ESR spectrometers, the RE Series, designed for ultra-high sensitivities and long term stability. With very high sensitivities of $10^{10}$ spin/0·1 mT, the ESRs have wide applications in biological, pharmaceutical and medical research.

To achieve long-term stability and high power, Jeol have incorporated a solid oscillation element, a Gunn diode, as the microwave power source. Its durability, 20 000 hours or more, can be six times that of the alternative Klystron oscillator.

The ESR's standard cavity is cylindrical and allows the sample to be irradiated directly. It has a high Q value of 18 000 and operates in the TE O11 mode. This enables a very high signal/noise ratio of 460 : 1 to be achieved which is especially effective in examining low concentrations.

The RE Series offers a choice of magnets with pole diameters of 150, 180 and 300 mm. The data system computer simplifies setting up, recording and other procedures such as modulation frequency changes and otherwise cumbersome measurements of second harmonics.

Many options are available including cavities for high and low temperature working, for light irradiation, wafer measurement and light detection. Extensions are available for ENDOR experiments, spin-echo, rapid scan and Q band operation.

The first of the new RE Series to be brought into Europe is being installed at Royal Holloway and Bedford New College.

*For further information, contact: Jeol Ltd., Jeol House, Grove Park, Colindale, London NW9 0JN, UK. Tel.: 01 205 6376.*

### Perkin-Elmer Model LS-50 luminescence spectrometer

The PC-controlled Perkin-Elmer Model LS-50 is a versatile, high performance, scanning luminescence spectrometer, capable of measuring fluorescence, phosphorescence, chemiluminescence and bioluminescence data. The Model LS-50 has the ability to selectively analyse many different types of sample, and is designed for accuracy of measurement, convenience and ease of use.

There is a comprehensive range of sample handling accessories, many of which are computer-controlled to automate sample handling for a wide range of applications.

Instrument control and comprehensive data handling facilities are provided through the menu-driven, GEM-based software. The data format allows further processing on other commercially available PC programs when necessary.

The Model LS-50's high energy optical system gives excellent sensitivity for both routine and research applications, and the pulsed xenon source provides the flexibility for measuring the fluorescence and phosphorescence of samples.

*For further information, contact: Perkin-Elmer Limited, Post Office Lane, Beaconsfield, Bucks HP9 1QA, UK. Tel.: 049 46 6161.*

### CENELEC hazardous area approval received for process IR Analyser

Servomex have just received LCIE approval to CENELEC specifications EN50014 and EN50016 for their purged PSA402 process infrared analyser.

CENELEC EEx p approval allows the PSA402 to be used in Zone 1 and Zone 2 hazardous areas and with group II gases.

Gas, liquid and vapour measurements can all be performed by the 402 allowing components ranging from CO and $CO_2$ to $SO_2$, solvents, water vapour and water in solvents to be detected.

The single-beam dual wavelength technique used in this analyser provides users with a highly stable, accurate measurement which has a high tolerance to sample cell contamination.

The two unit design, where the optical bench is normally mounted near to the sampling point and the control unit is located in a more convenient position, allows separation by as much as 500 metres.

Approvals received are EEx p II T5 for the PSA402 (Cert No LCIE 88.B6088X) and EEx p II for its associated pneumatic puge controller (Cert No LCIE 88.B0019U).

*For further information, contact: Servomex Ltd., Crowborough, Sussex, TN6 3DU, UK. Tel.: 0892 652181.*

**Analysis methods for environmental control**

Radiometer have documented a series of 25 analyses for chemists concerned with water and environmental analysis.

Typical examples are: fluoride in water, nitrate in soil and vegetables and chemical oxygen demand in waste water. Also included are 'A quick guide to ion-selective electrode methods' and 'A quick guide to conductivity measurement.'

The 25 free environmental analysis are available as 'The Environmental Package' from Radiometer's Analytical Division.

*For further information, contact: Radiometer Ltd., The Manor, Manor Royal, Crawley, West Sussex RH10 2PY, UK. Tel.: 0293 517599.*

**1989 ASTM Directory of Testing Laboratories**

The 1989 Directory is 40% revised since the 1988 edition, and features 1100 laboratories. Each entry gives a contact name, phone number, address, speciality, field of testing covered, materials and products analysed, equipment and staff available.

The laboratories featured perform services for a fee. They are not certified or endorsed by ASTM; they are listed as a service to ASTM members and customers.

List price, $50.00; ASTM member price, $40.00. ISBN 0 8031 1212 2; Code number 04 333289 32.

*For further information, contact: ASTM, 1916 Race Street, Philadelphia, PA 19103, USA. Tel.: 215 299 5400. ASTM European Office, 68a Wilbury Way, Hitchin, Herts SG4 0TP, UK. Tel.: 0462 31525.*

---

### This space could be working for you!

**Why not book this space to advertise your products, books, conferences, seminars, or job vacancies?**

For a free sample copy and rate card please contact:

**UK**
Mary Messer
Advertising Manager
Taylor & Francis Ltd
Rankine Road
Basingstoke
Hants RG24 0PR

**USA**
Joyce Chin
Advertising Manager
Taylor & Francis Inc
3 East 44th Street
New York,
NY 10017

